# Psychosocial predictors of outcome: time to relapse and survival in patients with early stage melanoma

**DOI:** 10.1054/bjoc.2000.1471

**Published:** 2000-11-22

**Authors:** J E Brown, P N Butow, G Culjak, A S Coates, S M Dunn

**Affiliations:** Medical Psychology Unit, University of Sydney, NSW, 2006; Sydney Melanoma Unit, Royal Prince Alfred Hospital, Camperdown, 2151; The Australian Cancer Society and Department of Public Health and Community Medicine, University of Sydney, 2006

**Keywords:** psychosocial, predictors, relapse, survival, melanoma

## Abstract

This study explored psychosocial predictors of relapse and survival in early stage melanoma patients. Patients with locoregional melanoma whose tumour thickness exceeded 0.69 mm, seen at the Sydney Melanoma Unit between 1991 and 1996 participated in the study. Questionnaires were sent to participating patients every 3 months for 2 years. Domains measured included cognitive appraisal of threat, coping, psychological adjustment, quality of life and perceived aim of treatment. Disease and demographic data were obtained from medical records. Multivariate analyses from baseline data used the Cox proportional hazards model. Of the 682 patients invited to participate 426 (62%) agreed. 91 (21%) relapsed and 60 (14%) died within the follow-up period, that ended in October 1997. After controlling for known prognostic indicators, several psychosocial variables predicted time to relapse and/or survival duration. Patients who perceived their aim of treatment to be cured, who did not use avoidance as a coping strategy or who were concerned about their disease experienced longer periods without relapse. Shorter survival duration was associated with a positive mood, the use of avoidance as a coping strategy, not being concerned with their disease and concern about the impact of the disease on family. There is still much to learn about the potential relationships between psychological well being, human behaviours and cancer outcome. Research in this area needs to clarify the psychological processes, as well as understand the biological and/or behavioural mechanisms that may link them to outcome. © 2000 Cancer Research Campaign http://www.bjcancer.com


*British Journal of Cancer* (2000) **83**(11), 1448–1453
© 2000 Cancer Research Campaign
doi: 10.1054/ bjoc.2000.1471, available online at http://www.idealibrary.com on IDEAL®

http://www.bjcancer.com

### Psychosocial predictors of outcome: time to relapse and survival in patients with early stage melanoma

**JE Brown[1], PN Butow[1], G Culjak[2], AS Coates[3] and SM Dunn[1]**

[1]Medical Psychology Unit, University of Sydney, NSW 2006; [2]Sydney Melanoma Unit, Royal Prince Alfred Hospital, Camperdown, 2151; [3]The Australian Cancer Society and Department of Public Health and Community Medicine, University of Sydney, 2006

Summary This study explored psychosocial predictors of relapse and survival in early stage melanoma patients. Patients with locoregional melanoma whose tumour thickness exceeded 0.69 mm, seen at the Sydney Melanoma Unit between 1991 and 1996 participated in the study. Questionnaires were sent to participating patients every 3 months for 2 years. Domains measured included cognitive appraisal of threat, coping, psychological adjustment, quality of life and perceived aim of treatment. Disease and demographic data were obtained from medical records. Multivariate analyses from baseline data used the Cox proportional hazards model. Of the 682 patients invited to participate 426 (62%) agreed. 91 (21%) relapsed and 60 (14%) died within the follow-up period, that ended in October 1997. After controlling for known prognostic indicators, several psychosocial variables predicted time to relapse and/or survival duration. Patients who perceived their aim of treatment to be cured, who did not use avoidance as a coping strategy or who were concerned about their disease experienced longer periods without relapse. Shorter survival duration was associated with a positive mood, the use of avoidance as a coping strategy, not being concerned with their disease and concern about the impact of the disease on family. There is still much to learn about the potential relationships between psychological well being, human behaviours and cancer outcome. Research in this area needs to clarify the psychological processes, as well as understand the biological and/or behavioural mechanisms that may link them to outcome. © 2000 Cancer Research Campaign http://www.bjcancer.com

**Keywords:** psychosocial; predictors; relapse; survival; melanoma

There is some controversy regarding the hypothesis that psychosocial factors impact upon relapse and survival in cancer patients. Several prospective and intervention studies have found an association between psychosocial factors and outcome (Stavraky et al, 1988; Spiegel et al, 1989; Greer et al, 1990; Fawzy et al, 1993; Molassiotis et al, 1997; Butow et al, 1999). Other studies have failed to find any association (Jamison et al, 1987; Tross et al, 1996).

The reasons for these inconsistencies are complex, ranging from methodological problems in the research itself, to a lack of understanding about the psychological and/or biological processes that may be involved. For example, inadequate sample size and the omission of important prognostic indicators have been commonly cited as problematic (Mulder et al, 1992). Research in this area is also confused by the diverse array of study designs with differences in the psychosocial constructs measured, cancer types sampled, prognostic covariates included, type of analysis and timing of assessments. For this reason it is important to systematically explore differences between cancer stages and types using the same variables and study design.

The current study is part of a larger project examining the association between psychosocial factors and outcome in early and advanced stage disease for melanoma and breast cancer patients. We are now seeking to highlight the differences between metastatic and early stage melanoma through a comparison of the results. Psychosocial variables were selected to encompass a comprehensive model of response to illness. Previously Butow et al (1999) found that a range of quality of life variables, psychosocial variables, marital status and perceived aim of treatment predicted survival duration in a group of metastatic melanoma patients. Specifically, results showed that patients who were (i) more positive about their expected survival, (ii) minimized the impact of their cancer on their daily lives or (iii) who felt more angry, were more likely to have longer survival.

Few studies have specifically examined relationships between psychosocial factors and outcome in patients with early stage melanoma. Previous studies suggest that coping style may be important (Rogentine et al, 1979; Fawzy et al, 1993). Fawzy et al (1993) in a randomized controlled trial found that baseline coping (active-behavioural) and changes in coping were predictive of outcome. Rogentine et al (1979) found that non-relapsers reported a significantly higher effort to cope with their illness. Coping style has also been found to be associated with outcome in early stage breast cancer (Greer et al, 1990; Watson et al, 1999), although this association has not been found in European samples (Buddeberg et al, 1996; Giraldi et al, 1997).

The aim of this study was to explore the relationships between these psychosocial variables and relapse and survival in a group of patients with locoregional melanoma using a methodologically sound design and large sample size.

### PATIENTS AND METHODS

The study population consisted of patients with locoregional melanoma first seen at the Sydney Melanoma Unit between 1991

*Received 11 May 2000*
*Accepted 9 August 2000*

*Correspondence to:* PN Butow and 1996. Exclusion criteria included tumour thickness of less than 0.7 mm, time since diagnosis greater than one year, psychiatric illness and inability to speak English fluently.

Participating patients were asked to complete a series of questionnaires that were repeated every 3 months, where possible, for up to 2 years. However, for the purposes of this paper, data were taken from the first questionnaire only. A subsequent paper will explore the impact of longitudinal data on survival. Follow-up was terminated in October 1997 and data censored at that time.

Disease and demographic data were obtained from medical records. At the close of the study, disease status and outcome were determined through a search of computerized and paper-based medical records and, where necessary, by contact with the patient's general practitioner or the NSW cancer registry.

### Psychosocial assessment

Measures were chosen to systematically obtain data on the range of psychosocial factors thought to be associated with human responses to illness. The measures have proven psychometric status and applicability to cancer populations.

### *Cognitive appraisal of threat: independence, family and disease*

Cognitive appraisal is the process by which a person evaluates the implicit threat in a stressful encounter. We used the 13-item cognitive appraisal subscale of the Stress Questionnaire (Folkman, 1986). Each item has a 5-point Likert scale from 'worries me a lot' to 'does not worry me at all'. Factor analysis in a sample of 125 metastatic melanoma patients yielded 3 factors concerning independence, family and disease (Butow et al, 1999). Cronbach alphas for these 3 factors in this sample were 0.85, 0.79 and 0.69, respectively. Examples of these factors are 'that I will not be able to work' (Independence), 'that my family will be upset' (Family), 'that I will have to cope with a lot of pain' (Disease). Items were summed to a factor score and were linearly transformed to a scale of 0 to 100, with high scores indicating more concern/distress.

### *Coping: active, distraction and avoidant*

Coping is the process of managing demands (external or internal) that are appraised as taxing or exceeding the resources of a person. We used a self-report adaptation of the Weisman and Wordon General Coping Strategies Scale, the COPE, which measures the frequency of use of 15 problem-solving and emotion-focused coping strategies (Weisman and Worden, 1976–77). The COPE has been used successfully with cancer patients, and has been shown to be sensitive to age, diagnosis and staging of the illness. Patients indicated on a 5-point Likert scale the frequency with which they used each coping style (never to always). Factor analysis of the COPE in a sample of 125 metastatic melanoma patients yielded 3 factors: active, distraction and avoidant coping (Butow et al, 1999). Cronbach alphas for the 3 factors in this sample were 0.65, 0.51 and 0.24, respectively. Examples of these factors are 'seeking more information about the situation' (Active), 'trying to forget, putting it out of my mind, distracting myself' (Distraction) and 'drinking, eating or taking drugs to feel less tense' (Avoidant). Items were summed and linearly transformed to a scale of 0 to 100, with a high score indicating frequent use of that coping style.

### *Psychological adjustment: isolation, minimization and anger*

This concept is defined as the functional outcome of coping, including maintenance of emotional equilibrium, acceptance of the illness and maintenance of positive self-esteem. Psychological adjustment was measured using the 53-item Psychological Adjustment to Cancer scale (PAC), developed by Dunn et al (1997). The sensitivity of the PAC has been demonstrated in a randomized controlled trial assessing the impact of the use of the word 'cancer' and euphemisms for it (Dunn et al, 1993). Patients indicated on a 5-point Likert scale the degree to which they agreed or disagreed with each statement. A factor analysis in a sample of 125 metastatic melanoma patients resulted in a shorter 24-item, 3-factor scale, measuring stigma/isolation, minimization and anger (Butow et al, 1999). Cronbach alphas for the 3 factors in this sample were 0.81, 0.57 and 0.65, respectively. Raw scores were linearly transformed to a scale of 0 to 100. High scores indicate greater levels of isolation, minimization and anger. Stigma/isolation items included 'I try not to let people know about my cancer' and 'since getting cancer my body has felt unclean'. Minimization items included 'having cancer is not making any difference to my life at all' and 'the thought of having treatment does not worry me'. Anger items included 'cancer is the worst thing that has ever happened to me' and 'I am constantly asking myself the question – why me?'

### Perceived aim of treatment

Patients' perceived aim of treatment was measured at the initial assessment. Patients chose between 4 response options: complete cure, increased chances of long-term survival, increased chances of short-term survival or reduction of symptoms. In these analyses, patients who selected complete cure as their aim of treatment were compared with all other responses.

### Quality of life (QOL) indicators

Indicators of QOL were assessed using 3 single-item LASA scales measuring physical well-being, mood and perceived effort to cope (Priestman and Baum, 1976; Hurny et al, 1993). The scales were scored from 1 to 100 using a 100 mm line. Each scale is anchored at either end by phrases representing extremes of experience. Thus physical well-being is anchored by 'lousy' and 'good'; mood is anchored by 'miserable' and 'happy' and effort to cope is anchored by a 'great deal of effort' and 'no effort at all'. On each scale, a higher score reflects better QOL. These scales have beeen found to have adequate reliability and validity (Butow et al, 1991; Hurny et al, 1993; Bernhard et al, 1997).

### Disease and demographic variables

The following demographic and disease prognostic factors were recorded and included in analyses: age, gender, marital status (whether patients were in a relationship), time since diagnoses, tumour site and thickness, presence of ulceration, number of mitoses, and recent treatment received (Millar et al, 1998). Tumour sites were recorded into 4 variables. These were (i) legs/arms, (ii) head/neck, (iii) thorax and (iv) other. In this analysis, the absence of melanoma at a site is the reference for comparison with the other groups. Patients were also divided into those who had (i) no previous locoregional metastases (referent

© 2000 Cancer Research Campaign

**Table 1** Comparison between non-participants ($n$ = 256) and participants ($n$ = 426) on demographic and disease variables

| | Non-participants Mean (SE) | Participants Mean (SE) | *P* value |
|---|---|---|---|
| Variable name | | | |
| Time since diagnosis (months)[a] | 3.9 (0.1) | 3.3 (0.1) | <0.01 |
| Time to relapse (mean (years))[b] | 1.6 (0.2) | 1.7 (0.1) | 0.81 |
| Time to death (mean (years))[b] | 2.2 (0.2) | 2.92 (0.2) | 0.30 |
| Mitoses | 3.7 (0.0) | 4.5 (0.3) | <0.01 |
| Thickness | 2.6 (0.2) | 2.3 (0.1) | 0.11 |
| Age | 57 (1.0) | 53 (0.8) | <0.01 |
| | % (respondents) | % (respondents) | |
| Ulceration | 27 | 30 | 0.46 |
| Loco-regional metastases | 11 | 16 | 0.09 |
| Gender (males) | 60 | 61 | 0.73 |
| Marital status (in a relationship) | 70 | 73 | 0.40 |

[a]Time measured between diagnosis and date of invitation. [b]Time measured between date of invitation and relapse or survival.

group) and (ii) locoregional metastases including local/intransit tumours and delayed or synchronous nodes. The referent groups for gender, ulceration and treatment were female, no ulceration and no treatment.

### Statistical analysis

Relapse-free survival was measured from the time patients completed their first questionnaire (date of study entry) to their first relapse or to the date of censoring. Likewise survival was calculated as the time between the date of study entry and either death from melanoma or the censor date.

Data were analysed in a two-step process. First, the best subset of explanatory variables was selected according to the method of Lawless and Singhal (Lawless and Singhal, 1978). The best subset method is preferable to the stepwise method when there are many covariates, particularly when the covariates are inter-related (Ciampi et al, 1988). Once the best subset of variables was identified, multivariate analysis was conducted using Cox Regression Analysis. Variables included in the final model were tested using the likelihood ratio test ($\chi^2$ distribution, $P < 0.05$). All calculations were performed using the SPIDA statistical package (Gebski et al, 1992).

### RESULTS

426 of 682 eligible patients agreed to participate in the study. Participants and non-participants did not differ on gender ($\chi^2$ test, 0.12; $P = 0.73$), marital status ($\chi^2$ test, 0.73; $P = 0.40$) locoregional metastases ($\chi^2$ test, 2.87; $P = 0.09$). or tumour thickness ($t$ test, 1.58; $P = 0.11$). There was also no difference on time to relapse (Log-rank test, 0.01; $P = 0.99$) or survival duration (Logrank test, 1.97; $P = 0.16$) when measured from the time patients were invited to join the study. Non-participants were older ($t$ test, 3.73; $P < 0.001$) and had a greater number of mitoses ($t$ test, –3.23; $P < 0.001$). Distinguishing differences between participants and non-participants are summarized in Table 1.

Of the 505 participants, 79 were excluded from the sample as their time since diagnosis exceeded 1 year. From the final sample of 426, 91 (21%) patients relapsed and 60 (14%) patients died from melanoma within the follow-up period. The majority of the sample was Australian-born, male and married. Mean time since diagnosis was 4.5 months with only 6% of the sample entering the study within 2 months of diagnosis. Disease and demographic characteristics of the sample are shown in Table 2.

**Table 2** Demographic and disease characteristics of the final sample ($n$ = 426)

| | | Mean (SD) |
|---|---|---|
| **Age (years)** | | **53 (16)** |
| **Time since diagnosis to study entry (months)** | | **4.5 (2)** |
| **Tumour thickness (mm)** | | **2.3 (1.7)** |
| | | **%** |
| *Demographic* | | |
| Gender | Male | 61 |
| Education | Primary | 6 |
| | Intermediate | 56 |
| | HSC or equivalent | 13 |
| | Technical | 6 |
| | Undergraduate/postgraduate | 19 |
| Occupation | Managers/administrators/professionals | 42 |
| | Tradespersons | 13 |
| | Sales/personnel/clerks | 26 |
| | Machine operators/labourers | 16 |
| | Home duties | 3 |
| Ethnicity | Australian | 88 |
| Marital status | Single | 12 |
| | Married/de facto | 73 |
| | Separated/divorced | 9 |
| | Widowed | 6 |
| *Disease* | | |
| Ulceration | None | 73 |
| Primary site | Occult | 4 |
| | Legs/arms | 39 |
| | Head/neck | 16 |
| | Thorax | 40 |
| | Other | 1 |
| Loco-regional metastasis | No | 84 |
| Perceived aim of treatment | Complete cure | 84 |
| | Increased chances of long-term survival | 12 |
| | Reduction of discomfort | 1 |
| | Don't know | 3 |

### *Psychological data and QOL indicators*

Summaries of the psychological variables and QOL indicators for patient outcomes are presented in Table 3. This table suggests that the largest differences were found on the scales measuring patient concerns but overall differences between these patient groups were not very great. On average patients who relapsed or died reported using more active, distraction or avoidant styles of coping.

### *Relapse-free survival and overall survival*

Variables included in the multivariate analyses were selected using the best-subset method (Lawless and Singhal, 1978). Variables found to be significant in the multivariate analysis using Cox Regression Analysis are presented in Table 4.

While controlling for important prognostic indicators, it was found that patients who perceived their aim of treatment to be cure, who used less avoidance as a coping strategy and who were more concerned about their disease had longer relapse-free survival. 87% of patients who did not relapse perceived their treatment aim as complete cure, versus 71% of those who did relapse. The relapse-free survival graph for perceived aim of treatment is

 *© 2000 Cancer Research Campaign*

**Table 3** Descriptive statistics for psychological variables and QOL indicator variables

| | Non-relapsers Mean (Std Err) | Relapsers Mean (Std Err) | Died Mean (Std Err) |
|---|---|---|---|
| Psychological adjustment | | | |
| Isolation | 79 (0.7) | 78 (1.5) | 83 (1.7) |
| Minimization | 61 (0.9) | 62 (1.6) | 59 (2.2) |
| Anger | 38 (0.3) | 40 (1.7) | 35 (2.3) |
| Coping style | | | |
| Active | 45 (1.3) | 49 (2.2) | 51 (3.3) |
| Distraction | 33 (1.0) | 35 (1.9) | 36 (1.0) |
| Avoidance | 35 (0.7) | 38 (1.4) | 38 (1.9) |
| Concerns | | | |
| Independence | 17 (1.2) | 17 (2.7) | 16 (2.9) |
| Family | 24 (1.3) | 30 (3.5) | 32 (5.0) |
| Disease | 17 (1.0) | 13 (1.7) | 12 (1.7) |
| QOL Indicators | | | |
| Physical | 78 (1.4) | 77 (3.7) | 81 (3.8) |
| Mood | 77 (1.3) | 77 (3.5) | 81 (3.0) |
| PACIS | 79 (1.3) | 90 (1.7) | 85 (2.9) |

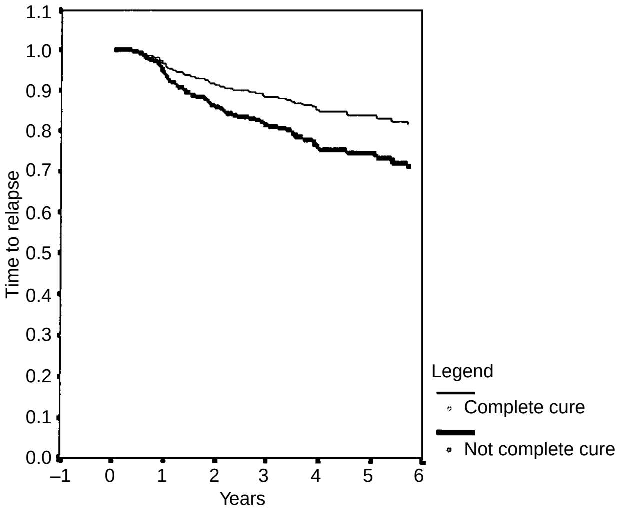

**Fig 1.** Survival graph for time to relapse for perceived aim of treatment.

shown in Figure 1. A different picture emerged if overall survival was taken as the outcome. Longer overall survival was associated with a negative mood, not using avoidance as a coping strategy, being concerned about the disease and not being concerned about its impact on the family.

### DISCUSSION

This study explored psychosocial predictors of outcome in 426 patients with early stage melanoma. While controlling for known prognostic variables, several psychosocial variables independently predicted time to relapse and/or survival duration. Those patients who used less avoidant coping strategies or who were more concerned about their disease overall, had a better outcome both in terms of time to relapse and survival duration. These findings are perhaps linked to preventative behaviours. By having a concern for their disease and not avoiding it, patients may be better able to look after themselves and engage in appropriate self-care, for example, have regular check-ups. Unfortunately, preventative behaviours were not monitored in the current study, so this link cannot be definitively established. Future research needs to identify and monitor other factors that may help establish these relationships.

Of the psychosocial variables, perceived aim of treatment had the greatest impact on time to relapse but was not associated with survival duration. Long-term associations may be weakened by the fact that some patients' perceptions about the treatment aim may not be stable over time, especially once the disease has spread. Unfortunately, this variable was only measured at baseline, so that conclusions about stability of this variable over time and situation cannot be made. Furthermore it is difficult to ascertain the degree to which patients' perception of their aim of treatment reflects a realistic understanding of their objective circumstanc or a specific 'optimistic/non-optimistic' psychological response or coping style. Further research is needed to clarify the factors influencing this perception, for example the degree to which patients base their expectations on the information provided by their treating physician.

Nevertheless, these results for perceived aim of treatment are consistent with those of Butow et al (1999) who found that

**Table 4** Final models for predictors of time to relapse and overall survival

| | Relapse | | | Survival | | |
|---|---|---|---|---|---|---|
| | *P* value | Hazard | 95% CI | *P* value | Hazard | 95% CI |
| Disease variables | | | | | | |
| Thickness | <0.001 | 1.33 | 1.19–1.49 | <0.001 | 1.34 | 1.17–1.53 |
| Ulceration | 0.005 | 2.03 | 1.24–3.31 | | | |
| Head/neck | | | | 0.02 | 2.63 | 1.19–5.78 |
| Thorax | | | | 0.07 | 2.01 | 0.95–4.24 |
| Prior loco-regional Metastasis | <0.001 | 4.32 | 2.60–7.18 | <0.001 | 3.54 | 1.83–6.83 |
| Perceived Aim of treatment | 0.06 | 0.59 | 0.34–1.02 | | | |
| Quality of life | | | | | | |
| Mood | | | | 0.06 | 1.02 | 1.00–1.03 |
| Coping style | | | | | | |
| Avoidant coping | 0.03 | 1.02 | 1.00–1.04 | 0.08 | 1.02 | 1.00–1.05 |
| Concerns | | | | | | |
| Family | | | | 0.03 | 1.01 | 1.00–1.02 |
| Disease | 0.008 | 0.98 | 0.96–0.99 | 0.01 | 0.97 | 0.95–0.99 |

*© 2000 Cancer Research Campaign*

perceived aim of treatment predicted survival in patients with metastatic melanoma. Thus where the disease is stable, this variable is associated with a significantly improved outcome in both early and late stage melanoma, supporting the need to further explore possible explanations for its impact.

Curiously, patient concern about family was found to have a hazardous effect on survival duration while patient concern about disease was found to be protective. This implies that these factors are not surrogates for a general measure of anxiety, but identify specific elements related to the cancer experience. Perhaps patients who are more concerned about their family experience signs and symptoms of worse disease. Alternatively, those concerned about their family may be more likely to direct their resources to their family and not to themselves. In any case, these results suggest that addressing issues about the impact of disease on both patients themselves and on their family could be beneficial for their overall outcome.

The findings of this study are consistent with other research reporting that coping style was associated with outcome in patients with primary melanoma (Fawzy et al, 1993). However, these results differ in terms of the specific coping styles found to be protective. For example, Fawzy et al (1993) found that an active coping style, and not an avoidant one, was associated with outcome. This discrepancy may be due to subtle differences in the items included within the measures. The low Cronbach alpha for the measure of avoidance in this study needs to be carefully considered. While the low alpha does not detract from the results themselves it may suggest that the items do not exclusively measure ‘avoidance’.

The relationship between mood and survival appears to be complex. There is some evidence to suggest that a negative ‘depressive’ mood is associated with a shorter overall survival (Faller et al, 1999; Watson et al, 1999). However, the general measure of mood used in this study may not reflect a ‘depressive’ mood but rather encompass other dimensions such as anger (Brown et al, in press). Anger has been shown to have a survival advantage in advanced stage patients (Butow et al, 1999).

While perceived aim of treatment has been found to be consistently associated with improved outcomes, other findings of this study differed from those of Butow et al (1999), even though the same variables were measured. For example, patients who minimized the impact of their cancer were found to have increased survival in patients with late stage but not early stage disease. Other research has also found that different patterns emerge when variables are measured at different times in the disease course. For example, Dean and Surtees (1989) found that outcome was associated with the use of stoic acceptance in the pre-operative period and denial in the postoperative period. Likewise Cassileth et al (1988) found differences in the pattern of results for early and late stage patients.

One explanation for these differences could be that different factors exert their influence at different stages in the disease course. The predictive capacity of biological variables is often limited to particular stage of the illness. For example, tumour thickness is a good predictor of an initial recurrence, but is not predictive of overall survival once a recurrence has occurred (Cohn-Cedermark et al, 1999). Dimensions of psychological experience may operate in a similar manner with certain dimensions being more prominent at one time versus another. It is apparent that patients’ psychological experience does vary across time (Heim et al, 1997). There are marked differences between early and late stage disease, for both physical experiences (e.g., surgery, chemotherapy) and existential issues that need to be faced (e.g., pain, death).

Overall, there is still much to learn about the potential relationships between psychological well being, human behaviours and the disease process. Research in this area needs to clarify the psychological processes themselves as well as to understand the biological and/or behavioural mechanisms that may link them to outcome.

### ACKNOWLEDGEMENTS

This project was supported by the National Health and Medical Research Council of Australia (Grant Nos: 910733, 940419). We also gratefully acknowledge additional funding provided by The Melanoma Foundation.

### REFERENCES

Bernhard J, Hurny C, Coates AS, Peterson HF, Castiglione-Gertsch M, Gelber RD, Goldhirsch A, Senn HJ and Rudenstam CM (1997) Quality of life assessment in patients receiving adjuvant therapy for breast cancer: the IBCSG approach. The International Breast Cancer Study Group [published erratum appears in Ann Oncol 1998 Feb9(2):231]. *Annals of Oncology* **8**: 825–835

Buddeberg C, Sieber M, Wolf C, Landolt-Ritter C, Richter D and Steiner R (1996) Are coping strategies related to disease outcome in early breast cancer? [see comments]. *Journal of Psychosomatic Research* **40**: 255–264

Brown JE, King MT, Buton PN, Dunn SM and Coates AS (2000) Patterns over time in quality of life, coping and pyschological adjustment in late stage melanoma patients: An application of multilevel models. *Quality of Life Research* **9**: 75–85

Butow P, Coates A, Dunn S, Bernhard J and Hurny C (1991) On the receiving end. IV: Validation of quality of life indicators. *Annals of Oncology* **2**: 597–603

Butow PN, Coates AS and Dunn SM (1999) Psychosocial predictors of survival in metastatic melanoma. *Journal of Clinical Oncology* **17**: 2256–2263

Cassileth BR, Walsh WP and Lusk EJ (1988) Psychological correlates of cancer survival: A subsequent report 3 to 8 years after cancer diagnosis. *Journal of Clinical Oncology* **6**: 1753–1759

Ciampi A, Lawless J, McKinney S and Singhal K (1988) Regression and recursive partition strategies in the analysis of medical survival data. *Journal of Clinical Epidemiology* **41**: 737–748

Cohn-Cedermark G, Mansson-Brahme E, Rutqvist LE, Larsson O, Singnomklao T and Ringborg U (1999) Metastatic patterns, clinical outcome, and malignant phenotype in malignant cutaneous melanoma. *Acta Oncologica* **38**: 549–557

Dean C and Surtees PG (1989) Do psychological factors predict survival in breast cancer? *Journal of Psychosomatic Research* **33**: 361–369

Dunn SM, Patterson P, Butow PN, Smartt HH, McCarthy WH and Tattersall MH (1993) Cancer by another name: a randomised trial of the effects of euphemism and uncertainty in communicating with cancer patients. *Journal of Clinical Oncology* **11**: 989–996

Dunn SM, Welch GW, Butow PN and Coates AS (1997) Refining the measurement of psychological adjustment in cancer. *Australian Journal of Psychology* **49**: 144–151

Faller H, Bulzebruck H, Drings P and Lang H (1999) Coping, distress, and survival among patients with lung cancer. *Archives of General Psychiatry* **56**: 756–762

Fawzy FI, Fawzy NW, Hyun CS, Elashoff R, Guthrie D, Fahey JL and Morton DL (1993) Malignant melanoma. Effects of an early structured psychiatric intervention, coping and affective state on recurrence and survival 6 years later. *Archives of General Psychiatry* **50**: 681–689

Folkman S (1986) Dynamics of a stressful encounter: Cognitive appraisal, coping, and encounter outcomes. *Journal of Personality and Social Psychology* **50**: 992–1003

Gebski A, Leung O, McNeil D and Lunn D (1992) *SPIDA Users Manual*. Statistical Computing Laboratory Pty Ltd: Sydney

Giraldi T, Rodani MG, Cartei G and Grassi L (1997) Psychosocial factors and breast cancer: a 6-year Italian follow-up study. *Psychotherapy and Psychosomatics* **66**: 229–236

 *© 2000 Cancer Research Campaign*

Greer S, Morris T, Pettingale KW and Haybittle JL (1990) Psychological response to breast cancer and 15-year outcome [letter]. *Lancet* **335**: 49–50

Heim E, Valach L and Schaffner L (1997) Coping and psychosocial adaptation: Longitudinal effects over time and stages in breast cancer. *Psychosomatic Medicine* **59**: 408–418

Hurny C, Bernhard J, Bacchi M et al (1993) The perceived adjustment to chronic illness scale (PACIS): A global indicator of coping for operable breast cancer patients in clinical trials. Swiss Group for Clinical Cancer Research (SAKK) and the International Breast Cancer Study Group (IBCSG). *Supportive Care in Cancer* **1**: 200–208

Jamison R, Burish T and Wallston K (1987) Psychogenic factors in predicting survival of breast cancer patients. *Journal of Clinical Oncology* **5**: 768–772

Lawless J and Singhal K (1978) Efficient screening of nonnormal regression models. *Biometrics* **34**: 318–327

Millar E, Cappelen-Smith C, Sarris M, Clarke R, Kearsley J and Lee C (1998) Prognostic factors in cutaneous malignant melanoma. *Cancer Forum* **22**: 178–180

Molassiotis A, Van Den Akker OB, Milligan DW and Goldman JM (1997) Symptom distress, coping style and biological variables as predictors of survival after bone marrow transplantation. *Journal of Psychosomatic Research* **42**: 275–285

Mulder CL, Van Der Pompe G, Spiegel D, Antoni MH and De Vries MJ (1992) Do psychosocial factors influence the course of breast cancer? A review of recent literature, methodological problems and future directions. *Psycho-Oncology* **1**: 155–167

Priestman TJ and Baum M (1976) Evaluation of quality of life in patients receiving treatment for advanced breast cancer. *Lancet* **1**: 899–900

Rogentine GN, Jr, van Kammen DP, Fox BH, Docherty JP, Rosenblatt JE, Boyd SC and Bunney WE, Jr (1979) Psychological factors in the prognosis of malignant melanoma: a prospective study. *Psychosomatic Medicine* **41**: 647–655

Spiegel D, Bloom JR, Kraemer HC and Gottheil E (1989) Effect of psychosocial treatment on survival of patients with metastatic breast cancer. *Lancet* **ii**: 888–891

Stavraky K, Donner A, Kincade J and MA, S (1988) The effect of psychosocial factors on lung cancer mortality at one year. *Journal of Clinical Epidemiology* **41**: 75–82

Tross S, Herndon J, 2nd, Korzun A, Kornblith AB, Cella DF, Holland JF, Raich P, Johnson A, Kiang DT, Perloff M, Norton L, Wood W and Holland JC (1996) Psychological symptoms and disease-free and overall survival in women with stage II breast cancer. Cancer and Leukemia Group B [see comments]. *Journal of the National Cancer Institute*, **88**: 661–667

Watson M, Haviland J, Greer S, Davidson J and Bliss JM (1999) Influence of psychological response on survival in breast cancer: a population-based cohort study. *Lancet* **354**: 1331–1336

Weisman A and Worden J (1976–77) The existential plight in cancer: significance of the first 100 days. *International Journal of Psychiatry in Medicine* **7**: 1–15

*© 2000 Cancer Research Campaign*
